# Editorial: Non-coding RNAs in breast cancer, volume II

**DOI:** 10.3389/fonc.2025.1561190

**Published:** 2025-02-11

**Authors:** Wenwen Zhang, Naoyuki Kataoka, Xiaoxiang Guan

**Affiliations:** ^1^ Department of Oncology, Nanjing First Hospital, Nanjing Medical University, Nanjing, China; ^2^ Laboratory of Cellular Biochemistry, Department of Animal Resource Sciences, Graduate School of Agricultural and Life Sciences, The University of Tokyo, Tokyo, Japan; ^3^ Department of Oncology, The First Affiliated Hospital of Nanjing Medical University, Nanjing, China; ^4^ Jiangsu Key Lab of Cancer Biomarkers, Prevention and Treatment, Collaborative Innovation Center for Personalized Cancer Medicine, Nanjing Medical University, Nanjing, China

**Keywords:** non-coding RNAs, breast cancer, ceRNA, therapeutic targets, biomarkers

Non-coding RNAs (ncRNAs) represent a category of RNA that do not possess protein-coding capabilities, encompassing microRNAs (miRNAs), long non-coding RNAs (lncRNAs), and circular RNAs (circRNAs). Despite their inability to encode proteins, ncRNAs play a crucial role in modulating the expression of various molecular targets, thereby influencing specific cellular biological processes and outcomes. To date, numerous ncRNAs have been identified and shown to be dysregulated across various cancer types, including breast cancer (1). Furthermore, the potential of ncRNAs as diagnostic and prognostic biomarkers and therapeutic targets has been thoroughly investigated in breast cancer (1, 2). This Research Topic collected 12 articles (four original research studies and eight reviews) that concentrated on novel discoveries or assessed recent advancements of ncRNAs in breast cancer.

Firstly, a comprehensive review by Li et al. highlights the promise of two significant classes of non-coding RNAs, namely lncRNAs and miRNAs, as both diagnostic and prognostic biomarkers for breast cancer. It also highlights them as potential targets for innovative therapeutic approaches. Chen et al. conducted a comprehensive review on the substantial impact of lncRNAs on the progression, diagnosis, and effectiveness of neoadjuvant chemotherapy in triple-negative breast cancer (TNBC), highlighting the varied expression profiles of dysregulated lncRNAs. They provided a summary of the precise mechanisms through which lncRNAs modulate gene expression in both the nucleus and cytoplasm, influencing post-transcriptional processes that affect mRNA stability and translation efficiency and thereby regulating tumor cell growth, proliferation, and metastasis.

ncRNAs have been reported to be involved in the regulation of signaling pathways for a variety of molecules or genes (3). 2-Methoxyestradiol (2ME2) is the primary endogenous metabolite of 17β-estradiol. It exhibits a diminished affinity for the estrogen receptor compared to 17β-estradiol and its derivatives, and its mechanism of action operates independently of the cellular response to estrogen (4). Subramani et al. found 2ME2 modifies the expression of pertinent genes by influencing the miRNome in TNBC cells, subsequently inhibiting TNBC through its impact on critical processes such as cell proliferation, apoptosis, and metastasis. Pyruvate kinase M2 (PKM2) serves as a crucial metabolic enzyme within the glycolytic pathway (5). Jemal et al. have conducted a comprehensive review of recent developments regarding the interactions of PKM2 with various transcription factors and proteins that influence the onset and progression of breast cancer. Additionally, they provide a summary of how natural compounds and non-coding RNAs modulate diverse biological processes in breast cancer cells by regulating the non-metabolic functions of PKM2. Moreover, Song et al. reviewed the localization, structural characteristics, and functional roles of various long non-coding RNAs (lncRNAs) linked to p53 pathway mechanisms or acting as transcriptional targets of p53, thereby enhancing our understanding of the interplay between lncRNAs and p53 in breast cancer. Zhao et al. reviewed the structural characteristics and mechanisms of action of lncRNA LINC00511, subsequently investigating its expression patterns and associated regulatory mechanisms in breast cancer. Furthermore, they examined the biological roles and prospective clinical implications of LINC00511 in breast cancer.

ncRNAs are also involved in the epigenetic regulation and drug resistance of breast cancer (6, 7). Yan et al. provided a comprehensive overview of the pivotal functions and intricate molecular pathways of lncRNAs in the emergence of tamoxifen resistance in breast cancer. Additionally, they evaluated the prospective clinical implications of lncRNAs as novel therapeutic targets and prognostic indicators in breast cancer. Wang et al. constructed an arginine methylation-associated lncRNA model and obtained an arginine methylation-associated lncRNA: lncRNA z68871.1. They demonstrated that the characterized lncRNA z68871.1 had a significant effect on the proliferation and invasion of breast cancer cells.

Single nucleotide polymorphisms (SNPs) are the predominant type of genetic variation. Investigating SNPs elucidates the variations in individual predisposition to diseases, disparities in pharmacological responses, and differences in reactions to environmental stimuli (8). The findings of Qi et al. confirmed that specific LncRNA H19 gene polymorphisms are linked to an increased susceptibility to breast cancer, and that the expression levels of associated genetic markers can profoundly influence the prognosis and therapeutic response in patients with breast cancer.

circRNAs are extensively investigated for their role as molecular sponges for miRNAs, which compete to bind to miRNA-targeted messenger RNAs (9). This interaction contributes to the establishment of a sophisticated post-transcriptional regulatory framework known as the competitive endogenous RNA (ceRNA) network (9). In this Research Topic, Liu et al. found EIF4A3 could enhance the expression of circ_0022382, and elevated levels of circ_0022382 may activate the PI3K/AKT/mTOR signaling pathway and SLC7A11 by sequestering let-7a-5p. Conversely, the silencing of circ_0022382 can impede the proliferation and migration of breast cancer cells, while also promoting the onset of disulfidptosis in breast cancer.

Exosomes represent a category of extracellular vesicles encased in a lipid bilayer, lacking intracellular organelles yet encompassing all known molecular components found within a cell. Tumor cells release exosomes approximately ten times greater than that of normal cells (10). These tumor-derived exosomes play a crucial role in mediating intercellular communication and are implicated in various phases of cancer progression (10). Primary breast tumor cells have been shown to sensitize brain microenvironmental cells, facilitating the development of breast cancer brain metastasis (BCBM) via the secretion of extracellular vesicle-associated miRNAs (11). miRNAs originating from breast tumors can also enhance the invasion of breast cancer cells across the blood-brain barrier by compromising the integrity of brain microvascular endothelial cells (11). Khan et al. provided a comprehensive review of the current literature on miRNAs derived from breast cancer that promote BCBM, detailing their roles in the intricate processes of BCBM, their interactions with microenvironmental cells within the brain metastatic niche, and discussing their potential therapeutic applications in the treatment of BCBM. Additionally, Blancas-Zugarazo et al. reviewed the insights into the cellular mechanisms modulated by exosomal lncRNAs that are critical for the development of chemoresistance and metastasis in breast cancer. They assessed the implications of utilizing exosomal lncRNAs as biomarkers for breast cancer in clinical settings, aiming to facilitate personalized patient management.

Collectively, all these studies in this Research Topic provide new perspectives on the function of ncRNAs in the development and advancement of breast cancer. Nevertheless, the potential of ncRNAs as therapeutic targets for breast cancer warrants further investigation in future studies. We anticipate that ncRNA-based therapies will soon emerge as feasible treatment alternatives for breast cancer patients, either as monotherapy or in combination with current therapeutic modalities.
